# Characterizing Spin in Psychiatric Clinical Research Literature Using Large Language Models

**DOI:** 10.1001/jamanetworkopen.2024.59500

**Published:** 2025-02-12

**Authors:** Roy H. Perlis

**Affiliations:** 1 Center for Quantitative Health, Massachusetts General Hospital, Boston; 2 Department of Psychiatry, Harvard Medical School, Boston, Massachusetts; 3 Associate Editor, *JAMA Network Open*

## Abstract

This quality improvement study describes the use of a large language model (LLM) to detect biased reporting in abstracts of psychiatric clinical research.

## Introduction

Spin, a form of biased reporting that presents study results more favorably than might an objective assessment,^1^ has been shown to be common in medical publications.^2^ Despite the attention this tactic has received, a recent study suggested rates of spin remain high.^3^ Because abstracts may be the primary means by which many clinicians interact with the medical literature,^4,5^ use of spin in abstracts may be particularly consequential. Readers of abstracts with spin are more likely to believe a study reported favorable results.^5^ Abstracts with spin risk distortion of prescribing practices or adoption of new technologies that may not be warranted by the evidence. As spin has received little attention in the psychiatric literature, I used a large language model (LLM) to develop an automated method to detect spin in psychiatric treatment abstracts.

## Methods

In this quality improvement study, I used the Bioconductor package to search PubMed for titles and abstracts of randomized clinical trials (RCTs) and meta-analyses of interventions published between January 1, 2013, and December 31, 2023, in the 3 highest-impact psychiatric journals: *American Journal of Psychiatry*, *Lancet Psychiatry*, and *JAMA Psychiatry* (eMethods in Supplement 1). In accordance with the Common Rule, this study was exempt from ethics review because it was not human participants research.

To characterize each abstract by presence or absence of spin, I applied a secure, private instance of an LLM (GPT4 Turbo) prompted by a definition^1^ and a set of categories^2^ of spin. After validation against gold-standard abstracts^5^ (eMethods in Supplement 1), the psychiatry journal titles and abstracts were presented (uploaded to LLM, June 15-16, 2024). Written permission to use these abstracts was provided by the journal publishers.

I used multiple logistic regression to examine whether journal, study type, intervention type, or publication year was associated with likelihood of including spin. Two-sided *P* < .05 indicated statistical significance. Analyses used R 4.3.2.

## Results

Sixty gold-standard abstracts, each scored 4 times, had 100% (95% CI, 98%-100%) sensitivity, 91% (95% CI, 86%-95%) specificity, and 95.8% (230 of 240) overall accuracy. Of the 663 abstracts identified, 296 (44.6%) exhibited possible or probable spin. Spin was likely in 230 of 529 RCT abstracts (43.5%) and 66 of 134 meta-analysis abstracts (49.3%) discussing medication (148 of 310 [47.7%]), psychotherapy (107 of 238 [45.0%]), and other interventions (41 of 115 [35.7%]). In a multivariable logistic regression model, reports of RCTs (odds ratio [OR], 0.58; 95% CI, 0.39-0.88) and nonpharmacologic or nonpsychotherapeutic interventions (OR, 0.63; 95% CI, 0.39-0.99) were significantly less likely to exhibit spin, as were reports with more recent publication (OR, 0.92; 95% CI, 0.87-0.97) (Figure).

**Figure.**
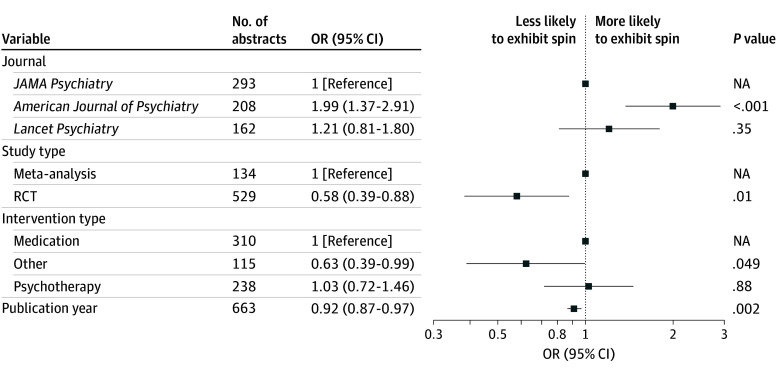
Logistic Regression Model of Abstracts Containing Possible Spin NA indicates not applicable; OR, odds ratio; RCT, randomized clinical trial.

## Discussion

In this application of an LLM to characterize spin in psychiatric abstracts, spin language was relatively common, particularly in meta-analyses, but diminished over time. One recent study of endometriosis RCTs found increasing rates of spin in abstracts over the past decade,^3^ whereas spin in systematic reviews of melanoma had modest diminution over time, although overall approximately 40% of abstracts reviewed included such language.^6^ I was unable to identify a prior report in psychiatry.

This work has limitations. While this novel application of LLM was validated, spin may have been overestimated given the imperfect specificity. These overestimates could reflect model hallucination or undetected biases; presenting abstracts in an interactive mode that uses the LLM’s chat capacity and allows further queries could improve interpretability (eMethods in Supplement 1). Alternative prompts or fine-tuning could also yield improved performance. Additionally, results were restricted to a subset of psychiatry journals; spin may be more prevalent in journals that publish clinical studies less frequently.

Nevertheless, the findings suggest that a substantial proportion of psychiatric intervention abstracts in high-impact journals present results in a potentially misleading way, which risks impacting clinical practice. Success in automating spin detection through LLM may facilitate identification and revision to minimize spin in future publications.

## Notes

Supplement 1.
eMethods

eReferences

Supplement 2.
Data Sharing Statement

## References

[1] Latronico N, Metelli M, Turin M, Piva S, Rasulo FA, Minelli C. Quality of reporting of randomized controlled trials published in Intensive Care Medicine from 2001 to 2010. Intensive Care Med. 2013;39(8):1386-1395. doi:10.1007/s00134-013-2947-3 23743522

[2] Yavchitz A, Ravaud P, Altman DG, . A new classification of spin in systematic reviews and meta-analyses was developed and ranked according to the severity. J Clin Epidemiol. 2016;75:56-65. doi:10.1016/j.jclinepi.2016.01.020 26845744

[3] Shirafkan H, Moher D, Mirabi P. The reporting quality and spin of randomized controlled trials of endometriosis pain: methodological study based on CONSORT extension on abstracts. PLoS One. 2024;19(5):e0302108. doi:10.1371/journal.pone.0302108 38696383 PMC11065215

[4] Barry HC, Ebell MH, Shaughnessy AF, Slawson DC, Nietzke F. Family physicians’ use of medical abstracts to guide decision making: style or substance? J Am Board Fam Pract. 2001;14(6):437-442. 11757886

[5] Boutron I, Altman DG, Hopewell S, Vera-Badillo F, Tannock I, Ravaud P. Impact of spin in the abstracts of articles reporting results of randomized controlled trials in the field of cancer: the SPIIN randomized controlled trial. J Clin Oncol. 2014;32(36):4120-4126. doi:10.1200/JCO.2014.56.7503 25403215

[6] Nowlin R, Wirtz A, Wenger D, . Spin in abstracts of systematic reviews and meta-analyses of melanoma therapies: cross-sectional analysis. JMIR Dermatol. 2022;5(1):e33996. doi:10.2196/33996 37632865 PMC10334896

