# Supplementary material for: Characterizing Spin in Psychiatric Clinical Research Literature Using Large Language Models
Source: JAMA Netw Open. 2025 Feb 12;8(2):e2459500. doi: 10.1001/jamanetworkopen.2024.59500 (PMC11822530; doi:10.1001/jamanetworkopen.2024.59500)
Supplement: Supplement 1. — eMethods eReferences [file jamanetwopen-e2459500-s001.pdf]

Supplemental Online Content

Perlis RH. Characterizing spin in psychiatric clinical research literature using large language models. *JAMA Netw Open*. 2025;8(2):e2459500. doi:10.1001/jamanetworkopen.2024.59500

eMethods

eReferences

This supplemental material has been provided by the authors to give readers additional information about their work.

© 2025 Perlis RH. *JAMA Network Open*

eMethods

Pubmed query:
(("JAMA Psychiatry"[Journal]) OR ("Am J Psychiatry"[Journal]) OR ("Archives of General Psychiatry"[Journal]) OR ("Lancet Psychiatry"[Journal])) AND (("clinical trial"[Publication Type]) OR ("meta-analysis"[Publication Type])) AND (2013/01/01:2023/12/31[PDAT]) AND (("treatment"[Title/Abstract]) OR ("medication"[Title/Abstract]) OR ("complementary medicine"[Title/Abstract]) OR ("ECT"[Title/Abstract]) OR ("TMS"[Title/Abstract]) OR ("ketamine"[Title/Abstract]) OR ("psychotherapy"[Title/Abstract]) OR ("intervention"[Title/Abstract]))

Large language model parameters and application:
GPT4-turbo (turbo-2024-04-09) without any fine-tuning or application of retrieval-augmented generation was applied with temperature set at 0 to yield results as close to deterministic as possible and maximize reproducibility, via Python script accessing the chat.ai application programming interface (API). We used a secure, private instance of GPT4 hosted by Microsoft Azure, which by contract does not allow the host to store materials or use them for future training purposes.

Model validation against gold standard:
We validated the prompt by presenting 30 gold-standard abstracts characterized as representing spin, and 30 edited to eliminate spin (reference #5), 4 times each in random order, to estimate sensitivity, specificity, and balanced accuracy.

Prompt to large language model (incorporates text adapted, summarized, and re-phrased from references 1 and 5; see main text for details):
Spin is defined as a way of reporting to convince readers that a treatment was beneficial, despite a statistically nonsignificant difference. Types of spin:
{Yavchitz et al (reference #1), Table 1, right column}

Statistically significant results should not be labeled as spin for categories focusing on reporting significance. A wide confidence interval is defined as crossing the null value by a substantial margin. A small effect size is defined as a clinically insignificant difference. Consider the context and full content of the abstract when identifying spin. Specifically:
- Sentences that indicate no statistically significant difference between treatment groups should not be considered spin for categories 6, 12, and 14.
- Sentences that provide statistically significant results with clear confidence intervals and p-values should not be considered spin for any category, especially for categories 6 and 12.
- Sentences that clearly and accurately report nonstatistically significant results should not be labeled as spin.
- Do not assign spin if #6 and/or #12 are the only concerning categories.

Now, for the following abstract, please only return a JSON object with 4 key-value pairs, with no other text: study design (RCT, naturalistic, meta-analysis); treatment studied (medication, psychotherapy, other); likelihood of spin (no, possible, probable) where possible is less than 50% confidence; sentence or phrase in abstract most worrisome for spin, if any; categories of spin in

© 2025 Perlis RH. *JAMA Network Open*

list form (#1-21 from the prompt). Example output: {"design": "RCT", "treatment":"medication","spin":"probable","sentence":"key sentence","categories":"[6,15]"} ...

Illustration of interactive application:

User: why is this spin?
ChatGPT (GPT4o):

The determination of spin in this abstract is based on the presence of several types of spin as defined in the prompt. Here's a breakdown of why it was labeled as "probable" spin:

1. **Selective reporting of or overemphasis on efficacy outcomes favoring the beneficial effect of the experimental intervention (#1)**:
   - The abstract emphasizes that [treatment] demonstrated antidepressant efficacy, highlighting the significantly greater improvement in HAM-D and HAM-A scores, while downplaying the fact that there was no significant improvement in the Visual Analog Scale for Pain score.
2. **Conclusion focusing selectively on statistically significant efficacy outcome (#6)**:
   - The conclusion claims efficacy based on statistically significant results for some measures (HAM-D and HAM-A), but does not adequately address the non-significant results for the Visual Analog Scale for Pain score or the 14% discontinuation rate due to intolerance in the [treatment] group.
3. **Conclusion claiming the beneficial effect of the experimental treatment despite high risk of bias in primary studies (#15)**:
   - The conclusion states that [treatment] demonstrated antidepressant efficacy without discussing the potential biases, such as the 14% discontinuation rate due to intolerance, which could impact the overall assessment of the treatment's efficacy and safety.

These factors contribute to the likelihood of spin, as they present the treatment in a more favorable light than might be warranted by the complete set of results.

User: Rewrite the conclusion to not exhibit spin

ChatGPT:

Certainly. Here's a revised conclusion that aims to present a balanced and comprehensive view of the study's findings:

Original Conclusion: "[Treatment] as an adjunct to escitalopram demonstrated antidepressant efficacy in adult patients with major depressive disorder experiencing persistent symptoms after 8 weeks of open-label treatment with escitalopram."

Revised Conclusion: "Adjunctive [treatment] to escitalopram showed significant improvement in depression severity as measured by the HAM-D and HAM-A scores in adult patients with major depressive disorder who had persistent symptoms after 8 weeks of open-label treatment

with escitalopram. However, there was no significant improvement in pain scores, and a notable percentage (14%) of patients in the [treatment] group discontinued treatment due to intolerance. These findings suggest potential benefits of adjunctive [treatment], but also highlight the need for careful consideration of its tolerability and the mixed results across different outcome measures."

eReferences

1. Yavchitz A, Ravaud P, Altman DG, et al. A new classification of spin in systematic reviews and meta-analyses was developed and ranked according to the severity. *J Clin Epidemiol*. 2016;75:56-65. doi:10.1016/j.jclinepi.2016.01.020

5. Boutron I, Altman DG, Hopewell S, Vera-Badillo F, Tannock I, Ravaud P. Impact of Spin in the Abstracts of Articles Reporting Results of Randomized Controlled Trials in the Field of Cancer: The SPIIN Randomized Controlled Trial. *JCO*. 2014;32(36):4120-4126. doi:10.1200/JCO.2014.56.7503
