# Supplementary material for: Characterizing Spin in Psychiatric Clinical Research Literature Using Large Language Models
Source: JAMA Netw Open. 2025 Feb 12;8(2):e2459500. doi: 10.1001/jamanetworkopen.2024.59500 (PMC11822530; doi:10.1001/jamanetworkopen.2024.59500)
Supplement: Supplement 2. — Data Sharing Statement [file jamanetwopen-e2459500-s002.pdf]

Data Sharing Statement

Perlis. Characterizing Spin in Psychiatric Clinical Research Literature Using Large Language Models. *JAMA Netw Open*. Published February 12, 2025.
doi:10.1001/jamanetworkopen.2024.59500

Data
Data available: Yes
Data types: Data (not involving human participants)
How to access data: Upon request of the author
When available: With publication

Supporting Documents
Document types: None

Additional Information
Who can access the data: qualified investigators
Types of analyses: for a specified purpose
Mechanisms of data availability: without investigator support
